# Bridging the gap in cardiovascular magnetic resonance imaging artificial intelligence implementations: From ambitious goals to real-world progress using foundation models

**DOI:** 10.1016/j.jocmr.2025.101979

**Published:** 2025-10-27

**Authors:** Neda Tavakoli, Amir Ali Rahsepar, Daniel Kim

**Affiliations:** Department of Radiology, Feinberg School of Medicine, Northwestern University, Chicago, Illinois, USA; Department of Radiological Sciences, Division of Cardiothoracic Imaging, David Geffen School of Medicine at UCLA, Los Angeles, California, USA; Department of Radiology, Feinberg School of Medicine, Northwestern University, Chicago, Illinois, USA

The use of deep learning models in cardiac imaging, including cardiovascular magnetic resonance (CMR) imaging, has the potential to enhance efficiency for cardiac imagers and generate more quantitative data compared to traditional qualitative assessments. However, integration of deep learning into cardiac imaging workflows remains fragmented. Clinicians and researchers currently rely on separate models for ventricular segmentation, tissue characterization, functional assessment, and disease detection, each of which requires costly expert annotations and task-specific development. Recently introduced advanced deep learning foundation models offer a potential solution. These large neural networks can be pretrained on diverse datasets and adapted to multiple downstream tasks with minimal additional training. This paradigm has already transformed natural language processing and is beginning to show promise in medical imaging, with models such as segment anything model (SAM) [1] and self-distillation with no labels version 2 (DINOv2) [2] providing broad cross-domain versatility.

Jacob et al. [3] present the first cardiac magnetic resonance (CMR)-specific foundation model trained on 36 million images across multiple sequences and evaluated on 9 clinical tasks. Jacob et al. [3] trained a vision transformer using self-supervised learning on CMR data from 27,524 subjects, including short- and long-axis cine, late gadolinium enhancement (LGE), parametric mapping, and flow imaging from UK Biobank and two clinical centers. After pretraining without labeled data, they adapted this single model to nine tasks spanning the typical CMR workflow as follows: classifying image types and cardiac views, segmenting ventricles and myocardium across different sequences, localizing anatomical landmarks, and detecting pathology. The results show consistent but modest improvements over a ResNet50 baseline [4]. Segmentation accuracy improved by 0.1–1.8 percentage points, improvements that, while statistically significant in some cases, hover near the threshold of clinical relevance. Cine view classification improved by 6.8 percentage points. More substantial gains appeared in the following data-limited scenarios: 3.7–6.6 percentage points for LGE hyperenhancement detection and 14 percentage points for disease classification, though from a very low baseline.

The study's most valuable contribution may be its systematic comparison of training strategies rather than its absolute performance gains. Vision transformers pretrained on natural images performed poorly on CMR tasks, sometimes worse than conventional convolutional networks. CMR-specific pretraining produced gains up to 70.7 percentage points points for aorta view classification and 9.1 percentage points points for LGE myocardium segmentation. This fundamentally challenges the assumption that general-purpose artificial intelligence (AI) models will readily transfer to medical imaging, showing that architectural flexibility becomes a liability without appropriate domain-specific pretraining.

While Jacob et al. [3] lay important groundwork for foundation models in cardiac imaging, their approach has several notable limitations. Disease detection accuracy reached only 70 percentage points, significantly lower than the 86–96 percentage points reported by task-specific models [5], [6]. While the authors attribute this to using only three frames from a single basal slice, this limitation highlights the following broader issue: foundation models alone may be inadequate for complex diagnostic tasks. For cardiomyopathy classification, where accurate interpretation requires integration of wall motion, volumetric analysis, and tissue characterization, the current approach falls substantially short. The study lacks true zero-shot capability, each task required fine-tuning on labeled data. While fewer examples were needed than baseline approaches, examples were still required. Deploying to new tasks or institutions still necessitates collecting and annotating training data, substantially limiting the practical efficiency gains that motivate foundation model development. Generalization testing was incomplete. Training on UK Biobank (Siemens 1.5T) and testing on the Automated Cardiac Diagnosis Challenge [ACDC] (Siemens 1.5T/3T) showed reasonable performance, but both datasets used the same vendor. Multi-vendor generalization across GE, Philips, Canon, United Imaging, and Siemens systems remains unproven, a critical requirement for real-world deployment that the field cannot afford to overlook.

Statistical considerations complicate interpretation. Some tasks used only 15–50 patients, raising questions about robustness. Many improvements did not reach statistical significance, such as mapping segmentation (gains of only 0.1–0.2 percentage points), LGE segmentation (gains of 0.1–1.8 percentage points), both LGE hyperenhancement detection tasks (despite 3.7–6.6 percentage points gains), and landmark localization (where one landmark improved by 0.1 mm while the other worsened by 0.1 mm). The clinical meaningfulness of 0.1–0.2 percentage points improvements in segmentation metrics is debatable, particularly when inter-observer variability between expert annotators exceeds these margins [5]. The trained model cannot be released due to data agreements, a significant barrier to reproducibility and scientific progress. Replication requires seven days on eight high-end GPUs (NVIDIA Tesla H100 [80 GB], NVIDIA, Santa Clara, California). This computational burden means only well-resourced institutions can develop foundation models, potentially exacerbating existing inequities in access to AI.

Beyond these technical limitations, the study analyzes images in isolation from the clinical context. Real CMR interpretation integrates imaging findings with patient history, symptoms, electrocardiogram findings, laboratory values, and prior studies. Whether foundation models can effectively incorporate multimodal clinical data remains unexplored, yet this integration is essential for clinical decision support. The authors evaluated tasks independently rather than as integrated workflow components. Real CMR analysis requires the following coordinated steps: identify sequence, classify view, segment structures, quantify function, and assess pathology. Whether a unified foundation model outperforms specialized models chained together is unclear, yet this comparison defines the value proposition of the foundation model approach. Without this analysis, we cannot determine if the increased complexity is justified. Prospective clinical validation is entirely absent. Whether foundation models improve diagnostic accuracy, improve cardiac imager efficiency, decrease inter-observer variability, or change clinical management in real-world use remains unknown. The gap between improved Dice scores and improved patient outcomes is substantial and their relationship cannot be assumed to be linear.

These limitations become more apparent when considering concurrent work that takes alternative approaches. The concurrent publication of ScarNet [7] provides an instructive contrast. Tavakoli et al. [7] developed a foundation model specifically for LGE scar segmentation by fine-tuning MedSAM [8] with specialized attention mechanisms for a single task. Their results show higher performance, achieving a median Dice 0.912 for scar segmentation and concordance correlation coefficient of 0.995 for scar volume quantification, substantially outperforming both general foundation models and standard approaches. While Jacob et al. [3] reported a Dice of 0.884 for myocardium segmentation without scar results, ScarNet [7] achieved higher performance at both myocardium segmentation (Dice 0.961) and the more challenging scar segmentation task (Dice 0.912).

This comparison illuminates a fundamental tension in foundation model development for cardiac imaging that the community must confront directly. Should we pursue general-purpose models that perform adequately across many tasks, or task-specific models that excel at individual applications? ScarNet [7] showed that focused optimization yields superior performance for specialized clinical needs, while Jacob et al. [3] show that broad pretraining enables reasonable performance across diverse applications with less task-specific data. CineMA [9], trained exclusively on cine sequences, represents a middle ground as follows: sequence-specific but multi-task. It achieved comparable performance to Jacob et al. [3] on standard cine views but struggled with non-standard acquisitions not included in its training data. Together, these three approaches, general CMR (Jacob et al. [3]), sequence-specific (CineMA [9]), and task-specific (ScarNet [7]), suggest that optimal foundation model strategy may depend critically on clinical deployment context. Institutions performing high volumes of specific analyses may benefit from task-specific models, while those requiring flexibility across diverse applications might accept the performance compromises of general models. However, this pragmatic accommodation may be settling for less than what the field ultimately needs.

Future research must address several critical needs. First, incorporate comprehensive spatial-temporal information through 3D or video architectures that capture the dynamic nature of cardiac function. Second, explore multimodal models integrating imaging with clinical data, moving beyond image-only analysis. Third, conduct rigorous multi-vendor validation to ensure models generalize across the heterogeneous scanner landscape of clinical practice. Fourth, perform prospective clinical trials evaluating patient outcomes, not just technical metrics. Fifth, develop true zero-shot adaptation methods that eliminate or drastically reduce the need for task-specific labeled data. The field also needs infrastructure for sharing pretrained models. The inability to release models due to data agreements highlights a systemic problem requiring collaborative solutions. Consortia focused on developing and distributing pretrained foundation models, similar to initiatives in other domains, could accelerate progress while addressing reproducibility concerns.

In conclusion, Jacob et al. [3] convincingly showed that developing CMR-specific foundation models is feasible, offering data efficiency benefits and modest performance improvements across multiple tasks. Their systematic ablation analyses provide meaningful insights into the value of domain-matched pretraining, the architectural needs of vision transformers, and the advantages of multi-sequence training, marking a genuine step forward in understanding how foundation models can be designed for cardiac imaging. However, positioning this as a clinically ready solution would be premature. The observed performance gains are incremental for most applications, and considerable gaps remain in disease detection capability, zero-shot learning, multi-vendor robustness, and multimodal integration. Compared with highly optimized task-specific models such as ScarNet [7], which achieve superior diagnostic performance through focused design, the practical benefits of general-purpose foundation models over specialized approaches remain uncertain. As the field progresses, maintaining realistic expectations while pursuing the ambitious goal of broadly applicable, clinically impactful foundation models will be essential. The work by Jacob et al. [3] represents an important first step, but the substantial technical, clinical, and infrastructural challenges ahead will determine whether foundation models ultimately transform cardiac imaging practice or remain a compelling yet limited academic exploration.

## Declaration of generative AI and AI-assisted technologies in the writing process

During the preparation of this work, the author(s) used ChatGPT in order to check the grammar. After using this tool/service, the author(s) reviewed and edited the content as needed and take(s) full responsibility for the content of the publication.

## Declaration of competing interest

The authors declare the following financial interests/personal relationships which may be considered as potential competing interests: Neda Tavakoli reports financial support was provided by National Institutes of Health, Radiological Society of North America, American Heart Association. Other authors declare that they have no known competing financial interests or personal relationships that could have appeared to influence the work reported in this paper.
